# Conservative treatment with brace and exercise for hyperkyphosis: a retrospective observational cohort study

**DOI:** 10.1186/1748-7161-9-S1-O39

**Published:** 2014-12-04

**Authors:** Salvatore Minnella, Sabrina Donzelli, Monia Lusini, Fabio Zaina, Michele Romano, Alessandra Negrini, Stefano Negrini

**Affiliations:** 1 ISICO, Italian Scientific Spine Institute, Milan, Italy; 2 University of Brescia, IRCCS Don Gnocchi, Milan, Italy

## Background

There are different types of hyperkyphosis which require specific strategies of treatment but there is little evidence in regard to its conservative treatment.

## Aim of the study

This study aim to assess conservative treatment of idiopathic and Scheuermann’s kyphosis by brace and specific exercise

## Design

This is a retrospective observational study on consecutive outpatients from a prospective database started in March 2003

## Methods

### Setting

Outpatient tertiary referral clinic specialized in conservative treatment of spinal deformities.

### Participants

In December 2013, among all the patients below 18 years of age at first evaluation, present in the database, we selected those respecting the following inclusion criteria:

• diagnosis of idiopathic or Scheuermann’s kyphosis

• at least two clinical evaluations at the time of therapy start (T0) and stop (T1)

• spinal X-rays (lateral projection) at the time T0 and T1

### Treatments

All patients underwent conservative treatment with rigid braces, specific for hyperkiphosis, associated with specific exercises. The brace was prescribed for at least 18 hours a day at the beginning of therapy.

### Statistics

We compared clinical and radiographic variations between start and end of therapy. Primary outcome criteria: percentage of patients worsened (>5°), stable or improved. Secondary: thoracic kyphosis (TK), lumbar lordosis (LL), C7 and L3 distance from plumbline, sagittal index (SI), which is the distance from plumbline of C7 plus L3. We used descriptive statistics to point out average values of secondary outcome parameters and their standard deviation.

## Results

We included 35 patients, mean age 14.2±1.8 (19 males).

Mean duration of treatment was 3.06±1.03 years. In regard to our primary outcome we found 23 patients improved (66%), 8 stable (23%) and 4 worsened (11%).

Highly significant improvements were found for the main spinal parameters: TK reduced from 54.8±10° to 44.8±10° (p<0,001), LL from 55.1±8.28° to 50.9±9° (p=0.04), SI from 113.1±21mm to 89±30mm (p=0.003) and C7 from 58.6±12mm to 47.5±13mm (p=0,001); while L3 changed from 53±12mm to 47±13mm (NS).

## Conclusion

According to our results conservative treatment with a rigid brace and specific exercise is an effective therapy of hyperkyphosis. It can significantly change both clinical and radiological parameters restoring more physiological values.

